# Twin Domain Structure in Magnetically Doped Bi_2_Se_3_ Topological Insulator

**DOI:** 10.3390/nano10102059

**Published:** 2020-10-19

**Authors:** Jakub Šebesta, Karel Carva, Dominik Kriegner, Jan Honolka

**Affiliations:** 1 Department of Condensed Matter Physics, Faculty of Mathematics and Physics, Charles University, Ke Karlovu 5, 121 16 Praha 2, Czech Republic; 2 Institute of Physics, Academy of Science of the Czech Republic, Na Slovance 2, 182 21 Praha 8, Czech Republic; dominik.kriegner@tu-dresden.de (D.K.); honolka@fzu.cz (J.H.); 3 Institute of Solid State and Materials Physics, Technical University of Dresden, 01062 Dresden, Germany

**Keywords:** topological insulators, magnetic doping, defects, ab initio

## Abstract

Twin domains are naturally present in the topological insulator Bi${}_{2}$Se${}_{3}$ and strongly affect its properties. While studies of their behavior in an otherwise ideal Bi${}_{2}$Se${}_{3}$ structure exist, little is known about their possible interaction with other defects. Extra information is needed, especially for the case of an artificial perturbation of topological insulator states by magnetic doping, which has attracted a lot of attention recently. Employing ab initio calculations based on a layered Green’s function formalism, we study the interaction between twin planes in Bi${}_{2}$Se${}_{3}$. We show the influence of various magnetic and nonmagnetic chemical defects on the twin plane formation energy and discuss the related modification of their distribution. Furthermore, we examine the change of the dopants’ magnetic properties at sites in the vicinity of a twin plane, and the dopants’ preference to occupy such sites. Our results suggest that twin planes repel each other at least over a vertical distance of 3–4 nm. However, in the presence of magnetic Mn or Fe defects, a close twin plane placement is preferred. Furthermore, calculated twin plane formation energies indicate that in this situation their formation becomes suppressed. Finally, we discuss the influence of twin planes on the surface band gap.

## 1. Introduction

Some of the most characteristic representatives of topological insulators (TI) are three-dimensional compounds with non-trivial topology protected by time reversal symmetry (TRS) [1,2,3,4]. Although their bulk band structure contains a band gap, the surface of such materials hosts unique conductive states, which intersect in the so-called Dirac point possessing a linear dispersion [1,4,5,6,7]. The formation of metallic surface electron states originates from the occurrence of band inversion driven by the strong spin orbit coupling (SOC) [8,9], which leads to non-trivial band topology protected by the TRS. It ensures band crossing at high symmetry points of the Brillouin zone [10,11], while no extra crystal symmetry is required (compare e.g., topological crystal insulators [1,5]). A combination of strong SOC, brought about by the occurrence of heavy elements as e.g., Bi, Se or Te, and TRS leads to the spin polarization of surface bands [3,5,10]. Electrons occupying states in the proximity of the Dirac cone with an opposite momentum also possess opposite spins (so-called spin-momentum locking). It ensures e.g., the suppression of back-scattering and related outstanding surface transport properties [11,12].

In real applications, the influence of defects could be important, since they might significantly alter properties of the ideal matter. They could be varied intentionally by chemical doping. In the case of TIs, it includes particularly magnetic doping. It can lead to the breaking of TRS, and therefore it opens a surface gap [13,14,15]. Then, not only does a possible control of the surface conductivity appear due to increased surface scattering, but also it could bring about the occurrence of new unique phenomena e.g., quantum anomalous Hall effect (QAHE) [16]. Besides, native defects occur there, naturally. Their presence is hardly controllable and they might have a significant impact on physical properties [17,18] as well. There could exist several kinds of native defects depending on the actual compound and the growth process. These include twin planes (TP), which are the focus of this article.

**Figure 1:**
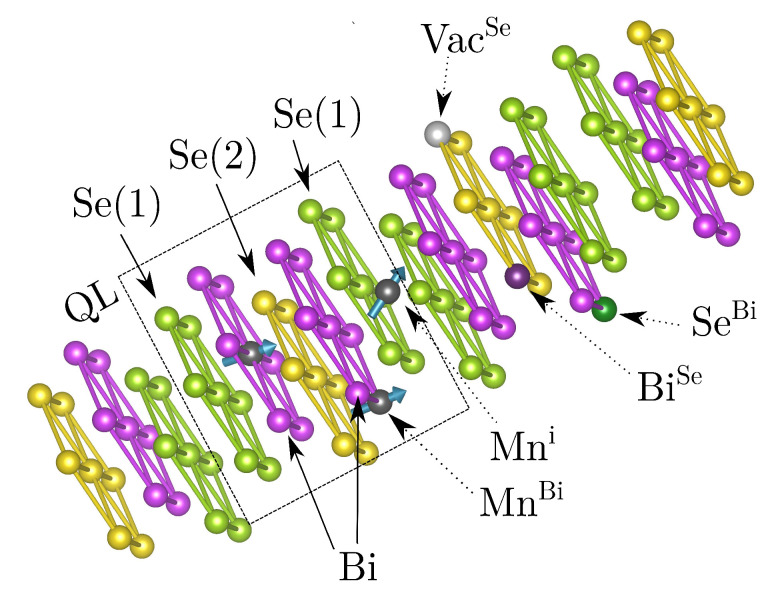
Layered crystal structure of Bi${}_{2}$Se${}_{3}$. Se and Bi layers gathered into QLs are depicted. Examples of (non)-magnetic defects are shown: (Mn${}^{\mathrm{Bi}}$) substitutional Mn atoms, (Mn${}^{i}$) interstitial Mn atoms, (Bi${}^{\mathrm{Se}}$) resp. (Se${}^{\mathrm{Bi}}$) Bi and Se antisites, (Vac${}^{\mathrm{Se}}$) Se vacancies.

**Figure 2:**
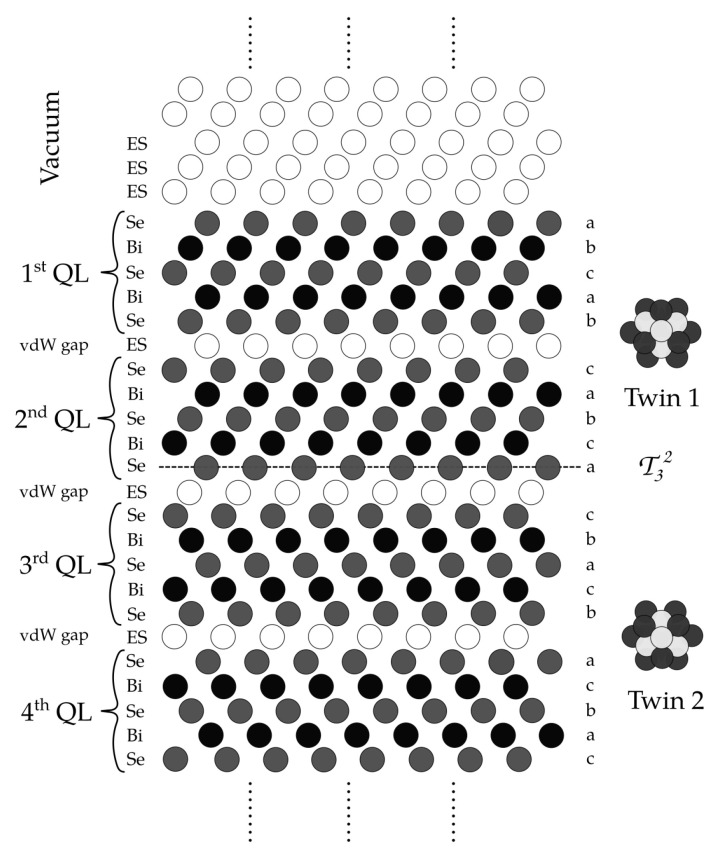
Layout of the simulated multilayer Bi${}_{2}$Se${}_{3}$ structure including twin planes. Proportions of atoms are not realistic within this schematic figure. QL—quintuple layer, ES—empty sphere, $T_{3}^{2}$ twin plane between the second QL and the third QL.

An important group of 3D topological insulators are bismuth chalcogenides, such as Bi${}_{2}$Se${}_{3}$ [8,19], which have been shown to be prone to the formation of twin domains [20,21]. This compound possesses a relatively simple band structure, convenient for experimental and theoretical studies, with a Dirac cone appearing at the Γ point. The crystal structure of Bi${}_{2}$Se${}_{3}$, belonging to the $R\bar{3}m$ space group, consists of Bi and Se hexagonal layers. These form quintuple layers (QLs) with alternating Bi and Se layers (Figure 1). Due to the coupling of QLs only by van der Waals (vdW) forces, there appears to be a gap between QLs, the so-called ‘van der Waals’ gap [19] (Figure 2). The gap consists of unoccupied tetrahedral and octahedral positions and its thickness is about 2.25 Å for Bi${}_{2}$Se${}_{3}$ [19]. The vdW gap allows cleaving the structure without breaking covalent bonds, which is important for the observation of topological surface states, as it does not yield any extra ingap states [22]. The presented crystal structure offers several sites, which could be occupied by magnetic atoms. Based on the theoretical and experimental studies, the most probable site for a magnetic dopant (Cr, Fe, or Mn) is the substitutional position, where magnetic atoms replace Bi ones [23,24,25,26,27]. Recently, a formation of septuple layers induced by magnetic defects was described. However, it was shown that it is negligible in Bi${}_{2}$Se${}_{3}$ for small concentrations of magnetic dopants [28]. Besides, there are studies which suggest an occupation of the interstitial positions within vdW gap concerning the related Bi${}_{2}$Te${}_{3}$ compound [29]. In our calculation, we especially employ Mn${}^{\mathrm{Bi}}$ [23,27,30,31] as well as Fe${}^{\mathrm{Bi}}$ [27,31,32] magnetic dopants. In addition in our calculations we also assume native defects like Bi or Se antisites (Bi${}^{\mathrm{Se}}$ resp. Se${}^{\mathrm{Bi}}$), where Bi atoms replace Se ones and vice versa. This non-stoichiometry arises due to difficulties in controlling growth conditions, which result in Bi- or Se- rich samples [23,33,34,35,36]. It has been shown that not only the magnetic defects, but also nonmagnetic disorder can substantially modify the dispersion of the Dirac surface states. [37]. Furthermore, regarding magnetically doped Bi${}_{2}$Se${}_{3}$, there exist suggestions that the surface band gap is not caused by magnetic ordering [38].

The above mentioned TPs represent a stacking fault of the layered structure of bismuth chalcogenides [20,21,39]. From symmetry arguments there are three three possible stacking positions of hexagonal layers alternating similarly to the fcc stacking sequence. During the formation of the crystal, there exist two energetically almost equivalent sites, which the atoms in the new layer can choose to occupy. Therefore, mirrored stacking might arise, which could be represented by a 60${}^{\circ }$ rotation of new layers in relation to the ideal ones [21]. It results in the inverse order of the *abc*-like stacking beyond the TP (Figure 2). Generally, there exist a few positions where TP could occur, but the most probable ones lie at outer chalcogenides of quintuples [40]. This means that stacking order inside each separate QL contains no defect; the perturbation occurs in the vdW gap between them. QLs after the TP are then constructed with a mirrored stacking order (Figure 2). The reported experiments show that the presence of TPs strongly depends on the used substrate [41,42].

Similarly to point defects, TPs might have a significant influence on the physical properties [43]. Therefore, in this paper, we focus on the influence of TPs on 3D TI Bi${}_{2}$Se${}_{3}$ behavior and their interplay with chemical disorder, especially the magnetic one. First, we describe a distribution of TPs in a nanoscopically thin Bi${}_{2}$Se${}_{3}$ slab. Then, we discuss their behavior under the presence of magnetic and nonmagnetic defects. Finally, the influence of TPs on the surface states is shown.

## 2. Methods

The study employs ab initio calculations done in the framework of the tight-binding linear muffin-tin orbital method within the atomic sphere approximation (TB-LMTO-ASA) formulated in terms of Green’s functions [44,45]. It involves the local spin density approximation with the Vosko–Wilk–Nursain exchange-correlation potential [46] and the use of a *s,p,d* atomic model. Calculations were treated in the scalar relativistic approximation, where on-site spin-orbit coupling was involved into the scalar relativistic Hamiltonian as a perturbation. An inclusion of spin-orbit coupling is needed to achieve a proper description of the electronic structure [5,8,47]. A basic screened impurity model was included to improve treating electrostatics of disordered systems [48]. Thanks to the use of the Green’s function formalism, chemical disorder could be included by the coherent potential approximation (CPA) [49]. It allows one to avoid using large statistical ensembles and it is suitable for small perturbation in the system. To simulate a layered structure with TPs, layered Green’s functions reflecting translation symmetry only within an atomic layer were employed [45,50,51,52]. In calculations, a multilayer system is attached to the semi-infinite leads, which have to satisfy self-consistent conditions. Due to the coupling of the multilayer to the attached leads, it is possible to obtain a self-consistent solution also for the inner layers. Based on the down-folding method, one is able to construct recursively embedding potentials acting from both sides on the particular layer, which are related to the interlayer coupling. For a detailed description, we refer the reader to Ref. [45,50].

The crystal structure is based on experimental Bi${}_{2}$Se${}_{3}$ lattice parameters ( unit cell *a* = 4.138 Å and *c* = 28.64 Å [19]), which were used to build Bi${}_{2}$Se${}_{3}$ multilayer structures. The vdW gap between QLs is included within ASA by placing appropriate empty spheres (ES). To avoid effects of the substrate (or leads) and to concentrate only on the behavior of proper Bi${}_{2}$Se${}_{3}$ layers, we surround it by vacuum, which is treated in a similar sense to the vdW gap. It is formed from the fcc-like stacked empty sphere layers keeping the three-fold symmetry of Bi${}_{2}$Se${}_{3}$ layers. Furthermore, because leads should fit to the simulated structure, slightly modified scandium is selected. Its hcp crystal structure suits fcc-like stacking within QLs and it possesses lattice parameters that are not too distinct [53]. However, leads are much less unimportant thanks to used vacuum spacers.

Finally, one is able to construct a layer structure, which consists of intermediate Sc layers at borders, coupled to semi-infinite leads, and several Bi${}_{2}$Se${}_{3}$ QLs enclosed by the vacuum spacer. In our calculation, we employed ten or twenty QLs wide Bi${}_{2}$Se${}_{3}$ structures and the vacuum spacers about ten ES layers width. These dimensions are sufficient to simulate the vacuum and to obtain surface gapless states. Native defects (Se${}^{\mathrm{Bi}}$), as well as magnetic doping by either Mn${}^{\mathrm{Bi}}$ or Fe${}^{\mathrm{Bi}}$, are included. In general, we assumed a homogeneous disorder, where mentioned defects occupy the appropriate sites with the same probability, unless otherwise stated. This assumption is supported by synchrotron experiments which show that Mn is not metallic in Bi${}_{2}$Se${}_{3}$ and thus does not segregate there [54]. The influence of the magnetic defects on the crystal structure is reflected by local lattice relaxation similar to the previous bulk calculation of Bi${}_{2}$Te${}_{3}$ and Bi${}_{2}$Se${}_{3}$ [17,30]. According to supercell calculations [30], we have modified and used the Wigner–Seitz radii locally while the total volume of Bi-sublattice is retained. Concerning details, we refer the reader to Ref. [30] and the Supplementary of Ref. [17]. Small changes which stem from the presence of the intrinsic Se${}^{\mathrm{Bi}}$ defects are neglected in correspondence to our previous work [30]. Besides, the relaxation corresponding to the presence of surfaces is not included there. In our calculation, we simulate TPs in the vdW gaps with respect to the required 2D periodicity in the layer. Hence no structure boundaries within a layer, which are related to the presence of TP [39], are involved.

The formation energy $E_{\mathrm{form}}^{\mathrm{def}(x)}$ of the extra stacking defect at *x* with respect to the unperturbed system is given as $E_{\mathrm{form}}^{\mathrm{def}(x)}=E_{\mathrm{total}}^{\mathrm{def}(x)}-E_{\mathrm{total}}$, where the energies on the right hand side correspond to total energies of the system with and without the defect. The selected approach unfortunately introduces numerical artifacts within the employed TB-LMTO-ASA framework, which renders calculations of the formation energy $E_{\mathrm{form}}$ not reliable. Therefore, we show only relative formation energies $\Delta E_{\mathrm{form}}$ considering the same number and similar type of stacking faults, while we focus on various composition and TPs distribution. These are defined so that zero energy level corresponds to a selected defect placement $x_{0}$, often with the lowest energy. Then

(1) $$\Delta E_{\mathrm{form}}^{\mathrm{def}(x)}=E_{\mathrm{form}}^{\mathrm{def}(x)}-E_{\mathrm{form}}^{\mathrm{def}(x_{0})}=E_{\mathrm{total}}^{\mathrm{def}(x)}-E_{\mathrm{total}}-\left(E_{\mathrm{total}}^{\mathrm{def}(x_{0})}-E_{\mathrm{total}}\right)=E_{\mathrm{total}}^{\mathrm{def}(x)}-E_{\mathrm{total}}^{\mathrm{def}(x_{0})}.$$

The energy differences w.r.t. the unperturbed system are thus canceled. Furthermore, the systematic error depends on the distance of the twin plane from the surface, as discussed in the Appendix A. The dependence obtained there is thus subtracted from data presented in Results where applicable. Since we deal with various positions of TPs within the multilayer sample, we describe their location by sub- and superscript, denoting adjacent QLs for clarity. The notation $T_{x+1}^{x}$ is used for simplicity, where QLs are enumerated from the top interface.

## 3. Results and Discussion

Stacking fault energies related to TP formation in ideal Bi${}_{2}$Se${}_{3}$ have already been studied elsewhere [39,40]. Here, we focus on their mutual interaction within Bi${}_{2}$Se${}_{3}$ slabs and subsequently on their interplay with chemical disorder.

### 3.1. Inter Twin Plane Interactions

#### 3.1.1. Structure without Disorder

**Figure 3:**
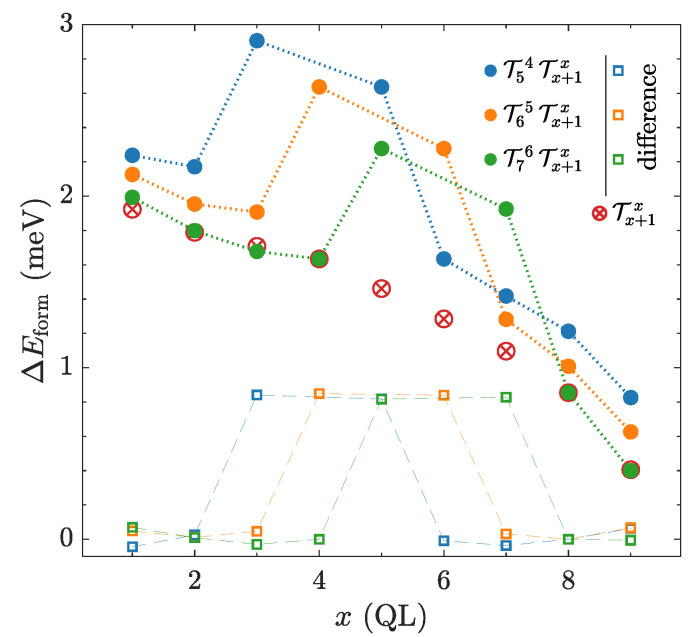
(●) Relative formation energy of two TPs as a function of their position within the structure, which consists of 10 Bi${}_{2}$Se${}_{3}$ QLs. (⊗) Relative formation energy of a single TP is depicted for comparison. Relative formation energies belonging to the different numbers of TPs are related to distinct absolute energies. (□) Energy curves associated to two TPs with subtracted single TP curve contribution.

One of the simplest ways to study interactions between TPs is the introduction of two TPs in the pristine Bi${}_{2}$Se${}_{3}$ multilayer structure. Keeping the position of one TP fixed while another one being independent allows us to determine corresponding relative formation energies over all possible mutual positions of TPs (Figure 3).

**Figure 4:**
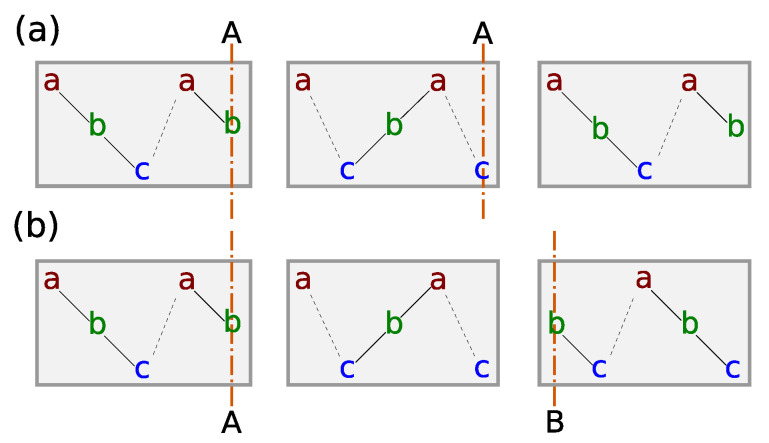
Twin plane orientation. There exist two mutual orientation of TPs. (**a**) TPs are located at the same sides of QLs (similar letters—AA resp. BB). (**b**) TPs occurs at the different sides of QLs (distinct letters—AB).

When studying multiple TPs, we have to consider that TPs can occupy the same or distinct sides of particular QLs (Figure 4). Different possible cases are compared in Section 3.4. In the remaining text, we show, for clarity, the simplest case, with identical orientations of TPs (denoted as AA according the Figure 4, resp. AAA in the case of two extra TPs). This situation represents qualitatively the most probable behavior, as it also resembles the lowest energy case of systems with non-identical TPs (Section 3.4).

**Figure A1:**
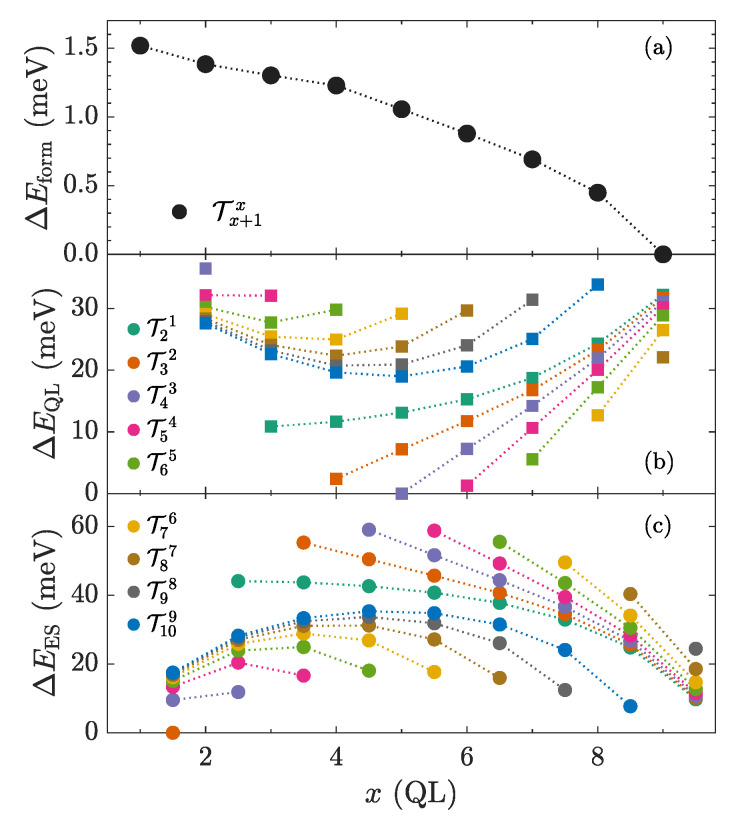
(**a**) Single TP relative formation energy $\Delta E_{\mathrm{form}}$ as a function of the TP position *x* in the Bi${}_{2}$Se${}_{3}$ multilayer. (**b**) Relative energy contribution $\Delta E_{\mathrm{QL}}$ of the particular QL *x*. (**c**) Relative energy contribution of ES as a function of their position. Bi${}_{2}$Se${}_{3}$ multilayer consists of 10 QLs. Concerning plots (b) and (c), dependencies belonging to several locations of TPs are depicted, where data points in the vicinity of TPs are excluded for clarity.

Considering two TPs in the multilayer structure consisting of 10 QLs, we find that the dependence related to a single TP, discussed in detail in the Appendix (Figure A1), is changed almost only for adjacent TPs, where an extra interaction energy appears (Figure 3). However, for such a small multilayer structure, it is not convenient to study TP interactions because of the strong interplay with the interfaces, which is shown in the Appendix (Figure A1a). It is reflected in the bending of calculated energy dependencies (Figure 3) caused by non-negligible energy contribution originating from the interactions between TPs and vacuum interfaces (Figure A1).

**Figure 5:**
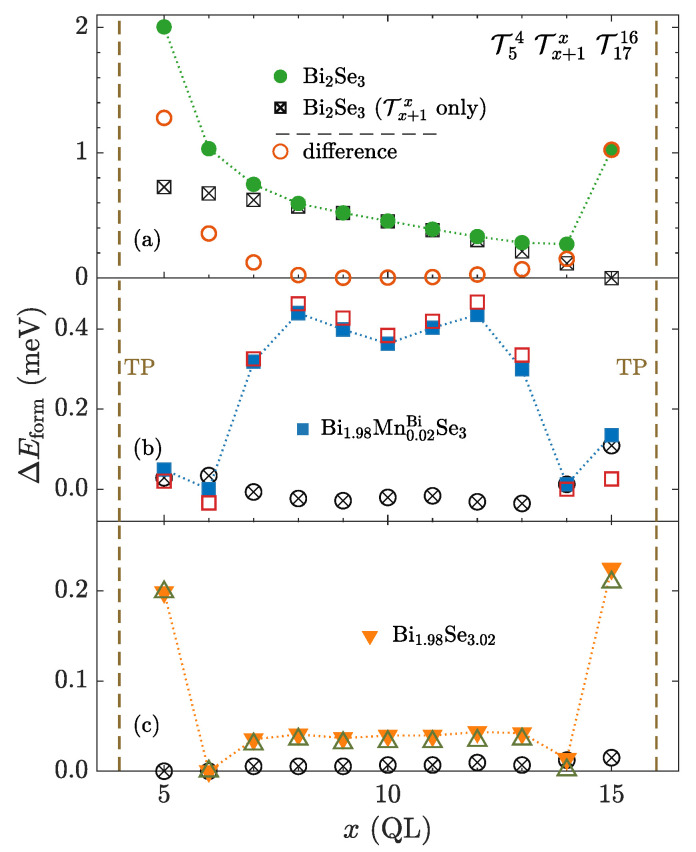
(●,⯀,▼) Relative formation energy of three TPs as a function of the position *x* of the middle TP. Positions of border TPs are fixed. The Bi${}_{2}$Se${}_{3}$ multilayer consists of 20 QLs. (**a**) pure system without any disorder. (**b**) system with homogeneous magnetic doping. (**c**) system under presence of homogeneously distributed nonmagnetic disorder. (⊗,⊠) Relative formation energy related to a single TP depicted for comparison. (◯,□,△) Energy curves associated to three TPs with a subtracted single TP curve contribution. Particular relative formation energy curves are related to different absolute energies.

Therefore, we introduce a larger structure, consisting of 20 Bi${}_{2}$Se${}_{3}$ QLs, where positions of two border TPs are fixed and the third one is able to move in between them (Figure 5a). This allows us to study the behavior of a TP in a more realistic situation, where it is affected primarily by other surrounding TPs rather than a surface. Interface proximity effects are thus reduced in this situation. The 3 TP calculation again shows a clearly visible repulsion of neighboring TPs (Figure 5a), especially after the subtraction of the surface-induced contribution to single TP energy (Figure A1). It reveals the occurrence of a significant interaction energy contribution appearing for TPs distant up to the length of three QLs. This suggests that TPs in a pure sample are likely spread over the sample with mutual distances which exceed at least the width of three or four QLs. Previous experiments utilizing X-ray nanobeam microscopy (D.Kriegner) [21] prove that if more TPs are observed, they are clearly several 10 nm apart. Hence, one can compare it with the width of one QL, which is about 1 nm [19]. This finding is supported by another experiment evidencing TPs separated by several QLs [20,41]. Nonetheless, one should be aware that we are comparing ground state calculations with a molecular beam epitaxy growth, which occurs far from equilibrium conditions.

#### 3.1.2. Native and Magnetic Defects

The interaction between TPs significantly changes when chemical disorder is introduced in the sample. For all studied types of doping (Mn${}^{\mathrm{Bi}}$ shown in Figure 5b, or Se${}^{\mathrm{Bi}}$ shown in Figure 5c), we observed a modification of the dependence of the relative formation energy on the distributions of TPs in comparison to the pure sample (Figure 5a). Due to the presence of disorder, the monotonous dependence on the distance from a certain vacuum interface disappears (compare Figure 5b,c to Figure 5a; ⊗-, ⊠-points). Moreover, the observed relative energy differences are almost one order of magnitude smaller (Figure 5b,c). It may be caused by suppressed interactions between TPs compared to the ideal case. The presence of a TP apparently does not represent such significant perturbation in disordered systems as it does in the case of pure, regular systems.

Calculations show that a system with magnetic disorder (Mn${}^{\mathrm{Bi}}$) prefers the gathering of TPs (Figure 5b) instead of their spreading observed in the undoped system. On the other hand, nonmagnetic disorder rather maintains a repulsion between TPs, although it is quite weak (Figure 5c) in comparison to the ideal case (Figure 5a), and it is non-negligible only for adjacent TPs. This indicates a significance of magnetism related effects, although we cannot exclude the role of different chemistry between dopant species. Therefore, we study the influence of the magnetism in more detail in the following sections.

### 3.2. Twin Plane Formation under Chemical Disorder

**Figure 6:**
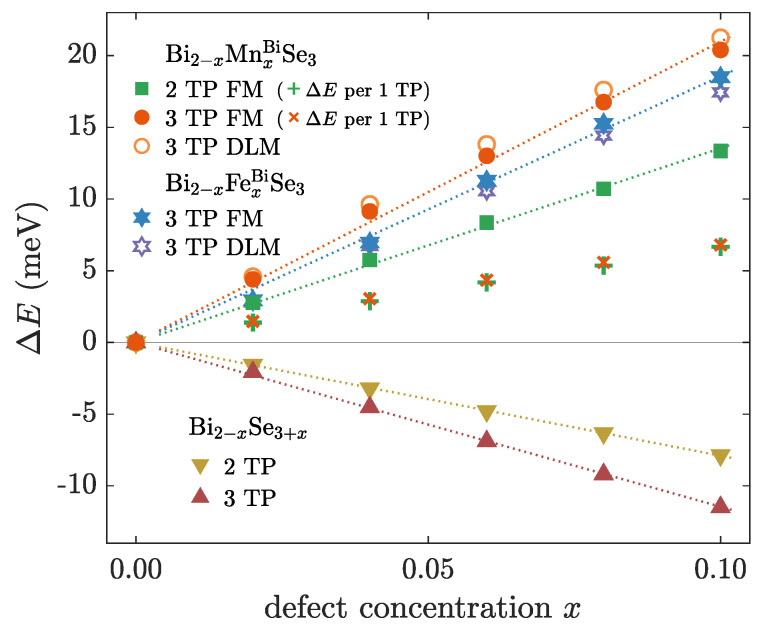
Relative change of the TP formation energy ΔE as a function of the concentration of defects *x*. Structures either with two ($T_{8}^{7}$, $T_{13}^{12}$ ) or three TPs ($T_{5}^{4}$, $T_{10}^{9}$, and $T_{17}^{16}$ ) within multilayers consisting of 20 QLs were used. Dependencies under presence of magnetic and native defects are depicted. (+) and (×) denote hypothetical relative formation energy related to a single TP. Dotted lines depict calculated linear fits.

We have calculated dependencies of the relative formation energy of TPs on the concentration *x* of magnetic Mn${}^{\mathrm{Bi}}$ or Fe${}^{\mathrm{Bi}}$ and native Se${}^{\mathrm{Bi}}$ defects to describe the influence of the defect presence on the tendency to TP formation (Figure 6).

The calculated relative formation energy, obtained for a different number of included TPs concerning also their distinct positions, almost linearly grows with the increasing concentration of magnetic defects. This proves that an increased amount of magnetic dopants *x* leads to the suppression of TPs in the multilayer. On the other hand, the nonmagnetic disorder (Se${}^{\mathrm{Bi}}$) decreases the relative formation energy of TPs in the structure. However, the appropriate dependencies exhibit linear behavior as well.

We assume that the suppression of TPs with respect to the increasing concentration of magnetic dopants corresponds to the observed tendency to gathered TPs in the case of magnetic doping (Figure 5b). We suppose that the gathering of TPs likely minimizes an induced effect on the electron structure, which arises from the interplay of TPs and disorder in connection with the magnetism. It agrees with the observation that TPs are less favorable in the magnetically doped systems (Figure 6). Analogously, the fact that the presence of nonmagnetic disorder does favor an occurrence of TPs (Figure 6) can be related to the suppressed impact of TPs on the system in that case (Figure 5c). Besides, one might note a proportionality of the relative formation energy to the number of the occuring TPs. It is confirmed by the comparison of the formation energy per single TP (Figure 6).

So far, we discussed ferromagnetically (FM) ordered magnetic dopants. Now, for a moment, we introduce a paramagnetic state represented by the disordered local moment (DLM) model, in order to decide whether the TP formation energy depends on the type of the magnetic order. In general, these two magnetic phases stand for the limiting cases of the magnetic order. One describes a perfectly ordered system, the other one an absolute disorder. One can observe that calculations exhibit only slight changes of the formation energy with a respect to the former FM order. It indicates that the formation energy likely hardly depends on the type of the magnetic order. More precisely, TPs become more favorable in the case of Fe doping. On the other hand, Mn doping illustrates an opposite behavior. We suppose that different slopes of energy dependencies induced either by Mn or by Fe dopants are likely related to different magnitudes of local exchange splitting. Calculations show that Fe atoms bear about $0.8\;\mu_{B}$ smaller magnitudes of magnetic moments than the Mn ones. Therefore, one might assume that the TP formation energy likely scales with the size of the change of the local exchange splitting caused by the mirrored crystal structure.

**Figure 7:**
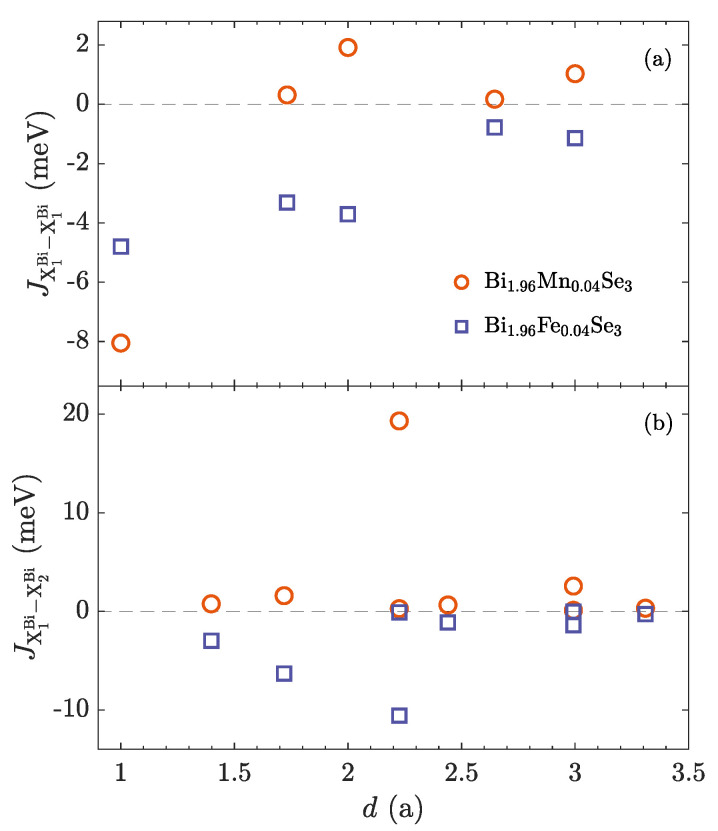
Exchange interactions between magnetic atoms at the substitution position within Bi${}_{2}$Se${}_{3}$ as a function of the distance in units of the lattice parameter *a*. Multilayered structures composed of 20 QLs and FM ordering are employed. Exchange interactions at central QLs are evaluated. (**a**) Exchange interaction within the same sublattice. Interactions within the atomic layer are depicted. (**b**) Exchange interaction between atoms occupying different sublattices. Interactions across the vdW gap are depicted only.

The mentioned quite large difference between the magnitudes of Fe and Mn magnetic moment stems from a distinct character of the magnetic exchange interactions (Figure 7), where they are evaluated by employing the Liechtenstein formula [30,55,56]. A comparison shows that unlike Mn related interactions, which are nearly positive except the nearest ones, the exchange interactions between Fe dopants are predominantly antiferromagnetic [57], regardless of the considered magnetic sublattices.

### 3.3. Magnetic Dopants Behavior

**Figure 8:**
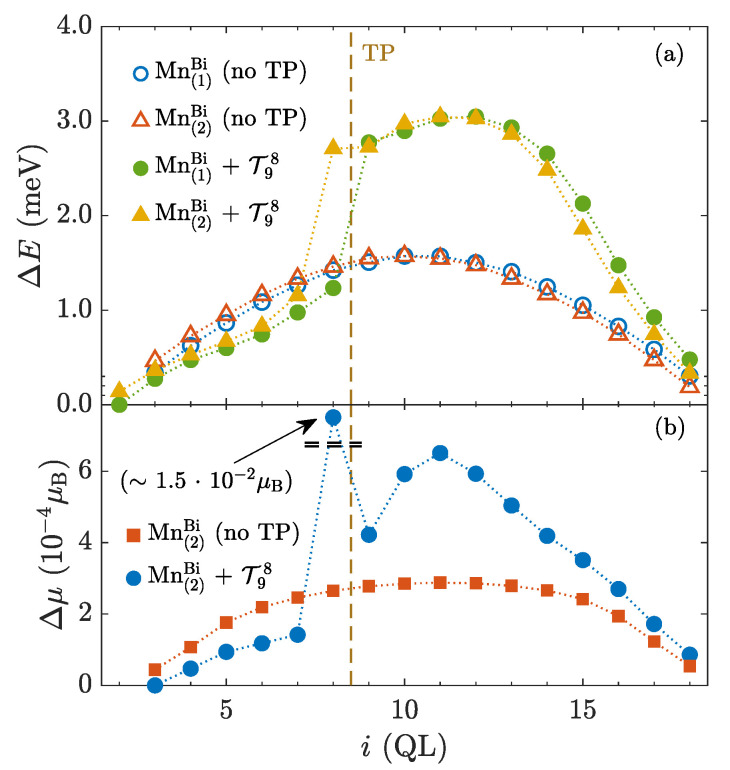
Magnetic doping of one layer inside the Bi${}_{2}$Se${}_{3}$ multilayer. (**a**) Relative formation energy of ${\mathrm{Mn}}^{\mathrm{Bi}}$ substition defects as a function of the doped QL *i*. ${\mathrm{Mn}}_{\left(1\right)}^{\mathrm{Bi}}$ and ${\mathrm{Mn}}_{\left(2\right)}^{\mathrm{Bi}}$ stand for substitutions at distinct Bi sites. ${\mathrm{Mn}}_{\left(1\right)}^{\mathrm{Bi}}$ faces neighbouring QL with lower *x*. Dependencies with and without TP are depicted. For clarity, they are shifted to fit at the end points. (**b**) Distribution of magnitudes of ${\mathrm{Mn}}^{\mathrm{Bi}}$ magnetic moments as a function of the position of the substitution *i*. Only one Bi site labeled by the index *i* of the appropriate QL is doped by 1% of Mn.

**Figure A2:**
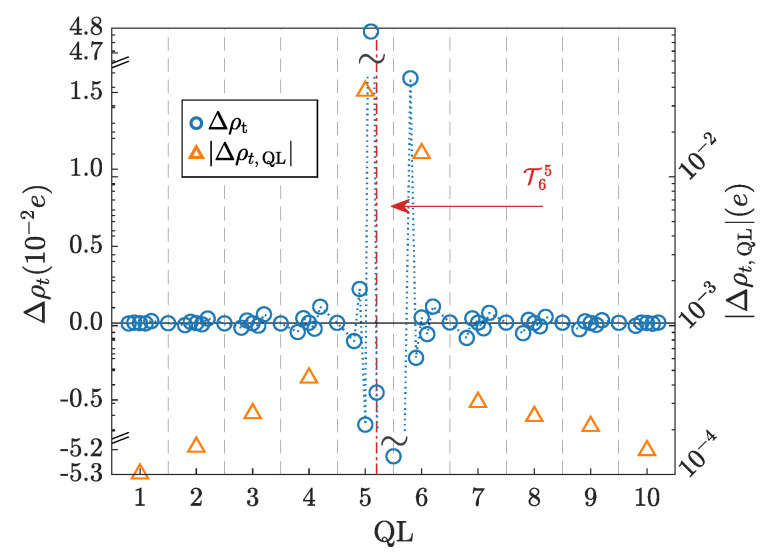
(left axis) Distribution of the charge transfer $\Delta \rho_{t}$ caused by the presence of a twin plane ($T_{6}^{5}$) in the multilayer composed of 10 Bi${}_{2}$Se${}_{3}$ QLs. Each data point denotes a particular atomic layer or an ES representing the vdW gap. (right axis) Dependence of the variance of the charge transfer at particular QLs. Dashed lines separate QLs and denote position of the vdW gap.

Next, we focused on the influence of TP on the behavior of magnetic dopants, and we calculated relative formation energies of Mn dopants as a function of the position of the dopant determined by indices of the Bi site and QL (Figure 8a). Only one Bi site in the whole structure is partly substituted by Mn. Comparing the shape of corresponding curves differing in the presence of a TP, one observes a clear variation of the relative formation energy caused by the TP. Dependencies without TPs bear a nearly symmetrical behavior, where a deviation is likely caused by an asymmetry of Bi sites concerning the QL structure. The occurrence of the TP modulates the shape of the former energy dependencies as particular sites become relatively more favored or disfavored according to their location with respect to the TP. A comparison of the formation energies (Figure 8a) with the magnitudes of induced magnetic moments on Mn dopants (Figure 8b) indicates a possible relation between the magnetism or spin splitting and the distribution of the relative formation energy of magnetic dopants. One can observe that the effectively suppressed relative formation energies, compared to the ideal structure, correspond to the weakening of induced magnetic moments, and vice versa (Figure 8b). A modulation of magnitudes of magnetic moments by stacking faults has been discussed e.g., in Pd films by means of ab-initio calculations [58]. The exceptional change of the magnitude of the magnetic moment, which is about two orders of magnitude larger than the other ones, stems from the proximity of the TP and it can be ascribed to the large charge transfer observed in the undoped structure, as described in the Appendix (Figure A2).

**Figure 9:**
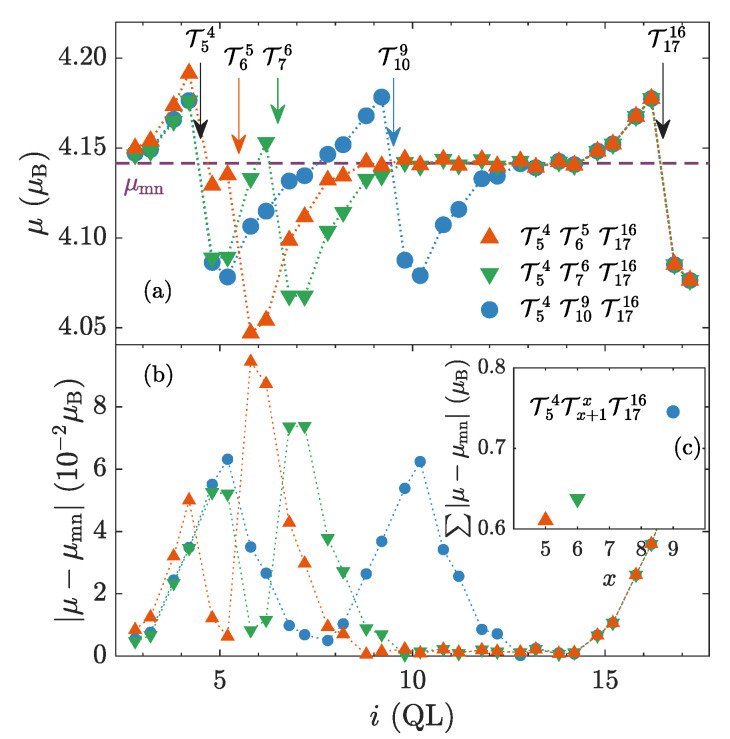
(**a**) Distributions of magnitudes of magnetic moments in homogeneously magnetically doped Bi${}_{1.98}$Mn${}_{0.02}$Se${}_{3}$ for various positions of TPs. ${\mathrm{Mn}}^{\mathrm{Bi}}$ are labeled by the index *i* of the appropriate QL. Arrows point to positions of introduced TPs. The outer TPs have fixed positions in all the cases (black arrows). Whereas, the inner one is being moved, the color of the arrow corresponds to the color of the function. (**b**) The absolute value of the change of the magnetic moment with the respect to the mean moment value $\mu_{\mathrm{mn}}$. (**c**) The sum of the magnetic moment changes from the previous subplot.

The described interplay of TPs and magnetic defects could explain the energetic gain observed for gathered TPs in a magnetic material (Figure 5). Close TPs lead to a smaller perturbation of the whole electronic structure. This might be deduced from the distribution of calculated magnitudes of magnetic moments in a homogeneously doped multilayer as a function of the positions of incorporated TPs (Figure 9a). We see that the closer TPs are, the smaller the overall variation of magnitudes of magnetic moments is (Figure 9c).

### 3.4. Comparison of Different TP Orientations

In the previous part, we described the simplest case consisting in the identical orientations of three TPs (AAA) (Figure 4). Now, we focus on the influence of nonidentical TPs on the preceding result. To examine it, we invert the orientation of each TP in the former three-TP structures, namely the orientations BAA, ABA, AAB are used (Figure 4), and we calculate the distribution of the formation energy.

**Figure 10:**
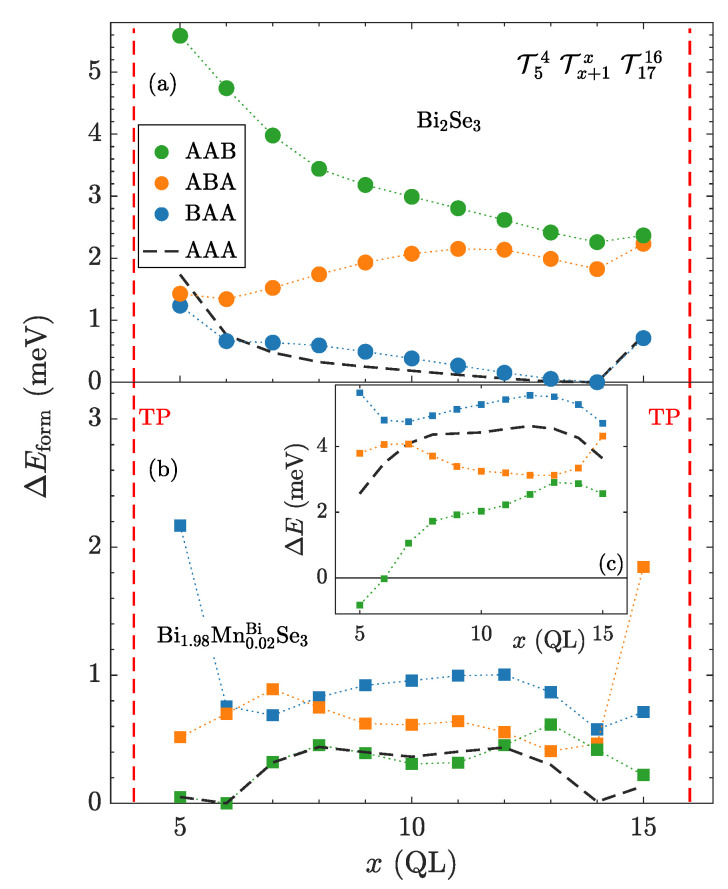
Dependences of the relative formation energies of three TPs on the position of the middle TP *x*. Different mutual orientations of the TPs are used. The orientation of TPs is labeled by letters A and B. (**a**) undoped structure. (**b**) magnetically doped structure. (**c**) change of the TP formation energy caused by presence of the magnetic defects—Bi${}_{1.98}$Mn${}_{0.02}$Se${}_{3}$ with respect to the undoped case. The relative formation energy curve related to identical TPs (AAA—dashed lines) depicted in panels (**a**,**b**) serves only as a shape reference.

**Figure 11:**
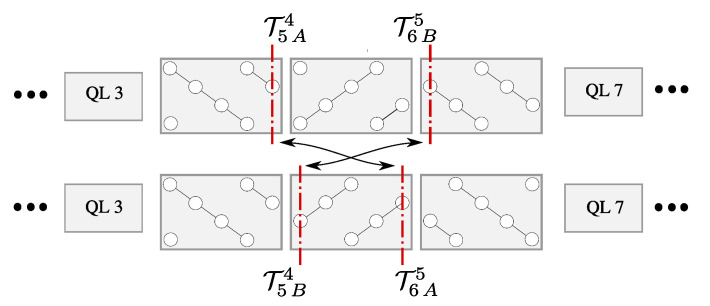
Sketch of the interchange of the AB TP order with the BA one in the case of gathered TPs.

We recall that in our approach, we are not able to compare the formation energies of the former identically oriented TPs with the case containing a single TP with the inverted orientation well. However, the mutual comparison of the new structures is feasible (Figure 10). Concerning the undoped structure, one can observe that, for the studied number of TPs, their formation energy strongly depends on the order of TP-type (Figure 10a). Considering an increasing index of QLs, it is evident that the BA order of two TPs, representing TPs at opposite QL sites (Figure 4), is more favorable than the AB one, which stands for TPs at an adjacent QL site. Namely, the BAA order, containing no AB sequence, has the lowest relative formation energy. Besides, it is clearly illustrated at “touching points”, where two adjacent TPs, either *A* or *B* type, are switching (Figure 10a), and the AB order with the BA one are interchanged (Figure 11). One can assume that the energy difference originates from the mentioned asymmetrical influence of TPs on the surrounding (Figure 8), which differentiate the AB and BA order. One can notice that the type of the TP sequence can be characterized by number of the vdW gaps in between TPs with respect to the system of identical TPs. According to the Figure 11), the AB segment contains an extra vdW gap, whereas the BA segment misses one.

Except the case of the inverted middle TP (ABA), the calculated energy curves qualitatively resemble the relative formation energy curve of identical TPs (Figure 10a). Namely, the BAA and AAA are nearly the same. The observed disfavor of the AB order likely gives rise to a slightly higher slope of the AAB energy curve in comparison to the case of identical TPs. The relative increase of the formation energy might be ascribed to elongation of the segment between *A*- and *B*-typed TPs. The shape of ABA formation energy curve can be explained in the similar way. There occurs a local maximum of the relative formation energy as a function of the position of the middle TPs. It likely originates from a complex interplay arising from the occurrence of two diametrically opposite inter-TP segments AB and BA, while their length is modified.

Considering the symmetry of the Bi${}_{2}$Se${}_{3}$ slab placed into the vacuum, where the BAA order is equivalent to the BBA one by a side inversion, one might assume that such immediate alternation of the TP types (ABA) is unlikely based on the calculated formation energies. Hence, it appears that the role of the TPs orientation can be regarded as marginal concerning the distribution of TPs in the undoped structure.

The relative formation energies dramatically change in the presence of the magnetic defects, similarly to the case of the identical TPs (Figure 10b). Although the energy curves are modified by the presence of distinct TPs, the local energy minima belonging to gathered TPs are kept. The presence of magnetic dopants reorders the formation energies according to the variation of the TPs orientation. It is likely related to the described interplay of TPs and the magnetic dopants (Figure 9). We show that the formation energy of TPs still grows with increased amount of the magnetic dopants, nearly irrespective of the TPs positions (compare Figure 10c and Figure 6). The existing exceptions originate from the special order of TPs, where TPs are more favorable under magnetic doping (Figure 10b). Besides, one should be aware that the calculated curves (Figure 10c) are influenced by vacuum interface-induced effects similar to the identical TPs (Figure 5). Finally, one can conclude that the TPs orientation does not cause significant qualitative changes in the TPs behavior, even in the case of magnetic disorder, as the lowest energy case almost mimics the behavior of the system with identical TPs.

### 3.5. Surface States

**Figure 12:**
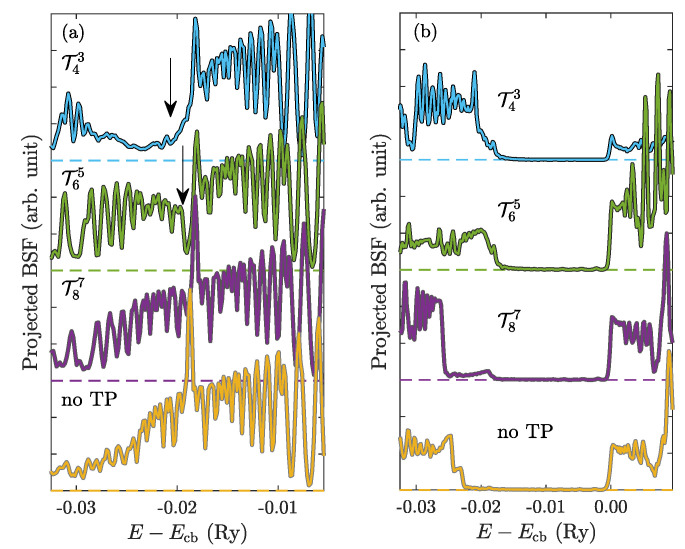
Projected BSFs of pure Bi${}_{2}$Se${}_{3}$ in the vicinity of Γ point as a function of the position of TP. Spin up and spin down channels are overlaped. Obtained surface gaps are denoted by arrows. (**a**) PBSF of the surface QL, (**b**) PBSF of the fifth QL from the surface. Energy axes are scaled to the position of conduction band edge $E_{\mathrm{cb}}$ at the fifth QL.

Conductive Dirac surface states are one of the most interesting properties of TIs. The appearance of TPs can strongly influence their presence, since the mirroring of the structure symmetry could represent a boundary in the structure. Hence, in this paper, we also try to simulate the influence of the presence of TP and its position on the surface states. We calculated Bloch spectral functions (BSF) in the vicinity of the Γ point, where the Dirac cone exists, on the path between high symmetrical reciprocal points *M* and *K*. In order to study the band gap and surface states, we project BSFs along the mentioned K−Γ−M path to the energy-intensity plane in a way that the maximal intensity of the BSF over the *k*-path is selected for particular energy points. Then, a formation of the Dirac states is indicated by the vanishing of the energy gap and an occurrence of a strikingly high intensity at the Dirac point, where the surface states intersects (Figure 12a). The projected BSFs (PBSF) reveal that the presence of a TP in at a certain distance from the surface breaks the surface Dirac states, which exist in the unperturbed structure (Figure 12a). Our calculations showed that, for TPs which are closer than 6 QLs to the surface, a gap opens, as seen most clearly in the presence of $T_{4}^{3}$ (Figure 12a).

The oscillations occurring in PBSF dependencies are caused by finite energy- and k-mesh, which prevents obtaining smooth electron bands in terms of the BSF as well as a narrow k-window, which cuts energy bands. Energy scales are related to the position of the well defined conduction band edge ($E_{\mathrm{cb}}$) at an inner QL (Figure 12b). As was mentioned, the observed canceling of surface states and gap opening likely arise from the proximity of two interfaces, which leads to a destructive interference [6]. Comparing results obtained for TP below the seventh QL with the unperturbed system, we found almost no difference as the Dirac cone is recovered. Similarly, one can mention a modulation of the bulk band gap width in the vicinity of a TP (Figure 12b), where a band gap width changes due to the presence of a boundary.

## 4. Conclusions

We have studied the behavior of TPs in the pure layered Bi${}_{2}$Se${}_{3}$ system, as well as in Bi${}_{2}$Se${}_{3}$ under the presence of magnetic and nonmagnetic disorder by first principles calculations. Our results show that interactions between TPs in the pure Bi${}_{2}$Se${}_{3}$ become negligible for distances above three QLs. However, for smaller distances, a significant increase of the TP formation energy was observed, in agreement with the experimentally observed spatial separation of TPs in real samples.

The distribution of TPs and their interplay significantly changes in the presence of chemical disorder. The presence of nonmagnetic disorder weakens the influence of TPs on the electron structure, and therefore the interactions between TPs are significantly smaller. However, the occurrence of magnetic defects modified the behavior of TPs significantly. Adjacent TPs become energetically more favorable, which corresponds to the dependence of the relative formation energy of TPs on the concentration of magnetic doping. It reflects a suppression of the TPs formation in magnetically doped structures, unlike nonmagnetic Se${}^{\mathrm{Bi}}$ antisites. The gathering of TPs leads to a smaller total perturbation of the electron structure and hence might be comprehended as a tendency to TPs annihilation. A thorough analysis indicates that the observed mismatch between the magnetic doping and the presence of TPs consists in the influence of TPs on the spin splitting of magnetic atoms.

On the other hand, the variation of spin splitting, caused by TPs, influences the site preference of magnetic defects. Mn generally does not prefer to occupy sites right at the twin boundary according to our calculations. Such behavior is indicated also in experiments, since no metallic Mn-Mn bonds were observed, although they would probably arise if clustering of Mn at these boundaries was present [54].

## Appendix A. Single TP Distribution

To check the behavior of a single TP in a pure multilayer structure, and to discuss the vacuum interface caused effect, a distribution of the single TP in a multilayer consisting of 10 QLs is studied. Thus, an asymmetric dependence of the relative TP formation energy as the function of the TP position was obtained (Figure A1a). According to the Figure 2, a TP occurs at Se sites in the vicinity of vdW gap. Generally, one is used to dividing a system to twin domains adjacent to the twin boundary. However, according to the structure composed of QLs, we hypothetically split the present system into two domains separated by a vdW gap. It seems to be more convenient to deal with entire QLs in energy comparisons. Nevertheless, one has to be aware that one of the domains contains a mirror layer. Since we used layer stacking according to Figure 2, a TP is located in the domain bearing smaller QL indices. The relative formation energy (Figure A1a) bears a monotonous dependence, which favors a width maximization of the domain, where the mirror layer belongs. It suggests a significant interplay of the TP with the surface interfaces, which depends on the orientation of the TP.

Naturally, the occurrence of a TP in the structure causes a charge transfer compared to the unperturbed system. Calculations show (Figure A2) that the electron density changes primarily in the QL possessing a mirror plane of the Bi${}_{2}$Se${}_{3}$ layer stacking. Especially, the charge is depleted from the vdW gap and it flows to the Bi layer adjacent to the TP located at Se sites. Besides, a TP causes charge oscillations, which spread out of the TP. Evaluating the absolute charge transfer $\sum |\Delta \rho_{t}|$ per a domain based on the charge transfer distribution, belonging to the system with symmetrical domain sizes (Figure A2); one finds that for the present way of stacking (Figure 2) the charge modulation is larger for the domain, consisting of QLs with smaller indices. The difference is about one order of magnitude, which implies a different impact on domains. Having also considered vacuum interfaces, which represent another structure fault, the system naturally should tend to a suppression of the energetically more demanding perturbations by their separation (Figure A1a).

Let us consider QLs and ESs separately and evaluate their relative total energy contributions. One observes that a clear discrepancy occurs between the two presented domains, regardless of the TP position. Relative magnitudes of total energies $\Delta E_{\mathrm{QL}}$ related to particular QLs *x* as a function of the position of the TP display that the domain containing the TP, namely QLs with the smaller indices, possesses higher $\Delta E_{\mathrm{QL}}$ and the closer a TP is to vacuum, the higher the magnitude of $\Delta E_{\mathrm{QL}}$. For brevity, we exclude QLs in the vicinity of the TP, since their relative energy changes are of a different scale. Similarly, relative energy contributions of ESs ($\Delta E_{\mathrm{ES}}$) are also influenced by the position of the TP (Figure A1c). However, they follow an opposite evolution compared to the energy contribution of QLs, likely because of an opposite impact of the stacking fault. The charge transfer of the related QLs and ESs differs in the sign. The formation energy curve (Figure A1a) includes both contributions, $\Delta E_{\mathrm{QL}}$ and $\Delta E_{\mathrm{ES}}$. However, we observe that the shape of the formation energy curve $\Delta E_{\mathrm{form}}$ (Figure A1a) is mostly determined by ES’s contribution $\Delta E_{\mathrm{ES}}$ (Figure A1c)

It is worth studying the influence of a single TP on surrounding atomic layers in a pristine structure, as it describes the bare effect of TP. It shows that the perturbation caused by a TP is hardly local. Dependencies of the charge transfer (Figure A2) or the QL resolved total energy (Figure A1c) indicate that the charge as well as energy modulation spread over few QL. Moreover, a strong interplay with the Bi${}_{2}$Se${}_{3}$ boundaries is visible, as it likely yields the shape of the $E_{\mathrm{form}}$ dependence (Figure A1a).
